# UPLC/Q-TOF MS Screening and Identification of Antibacterial Compounds in *Forsythia suspensa* (Thunb.) Vahl Leaves

**DOI:** 10.3389/fphar.2021.704260

**Published:** 2022-01-28

**Authors:** Mingyue Zhou, Jinhai Huo, Cairen Wang, Weiming Wang

**Affiliations:** ^1^ Institute of Chinese Materia Medica, Heilongjiang Academy of Chinese Medicine Sciences, Harbin, China; ^2^ State Key Laboratory of Quality Research in Chinese Medicines, Macau Institute for Applied Research in Medicine and Health, Macau University of Science and Technology, Taipa, China

**Keywords:** F suspensa leaves, chemical profiling, antibacterial mechanism, UPLC/Q-TOF MS, *S. aureus* and *E. coli*

## Abstract

*Forsythia suspensa* (Thunb.) Vahl (*F. suspensa*) is a traditional Chinese medical herb and only its fruit is currently used in clinical therapies. However, the discarded parts like leaves also contain a large number of active components. In this study, we used macroporous adsorption resin to enrich the effective components from *F. suspensa* leaves. The separated active compounds were then identified and quantified by ultra-performance liquid chromatography coupled with quadrupole time of flight mass spectrometry (UPLC/Q-TOF MS) and high-performance liquid chromatography Active components with antibacterial properties extracted from *F. suspensa* leaves were confirmed *in vitro* and the corresponding mechanisms were explored. In sum, a stable and effective method for extracting antibacterial active components from *F. suspensa* leaves was established in this study, which proved the practicability of *F. suspensa* leaves as traditional Chinese medicine and is conducive to the more comprehensive utilization of the plant.

## Introduction

**FIGURE 1:**
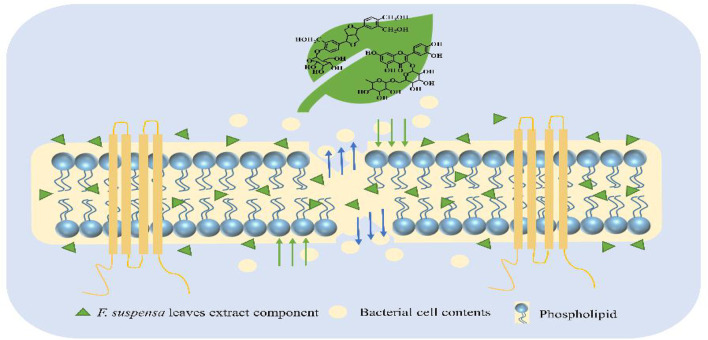
Antibacterial mechanism of *F. suspensa* leaves extract.

The fruit of *Forsythia suspensa* (Thunb.) Vahl (*F. suspensa*) has been widely used in clinical settings. However, the leaves of *Forsythia suspensa* receives little attention, in spite of their great medicinal value. Currently, *F. suspensa* leaves are mainly used for the treatment of pyrexia, inflammation, gonorrhea, carbuncle, and erysipelas. Modern pharmacological research has revealed that its pharmacological effects include anti-inflammatory, antioxidant, antibacterial, anti-cancer, anti-virus, anti-allergy, neuroprotective functions (Chen et al., 1999; Kim et al., 2003; Han et al., 2012; Lee et al., 2017; Wang et al., 2018). However, *F. suspensa* leaves are in large discarded during the harvesting process, which causes a waste of potentially valuable resources. It has been found that phenethyl alcohol glycosides are the main active components in the fruits of *F. suspensa* and play an antibacterial role, while the components in fruits and leaves are almost the same. In addition, the contents of forsythoside A, forsythin, and rutin in leaves are much higher than those in fruits, indicating that leaves could also possess an antibacterial effect *in vitro* (Tian et al., 2021). Thus, the by-products from the processing of *F. suspensa* leaves can play a key role in our daily lives, such as antibacterial hand sanitizer additives and health functional drinks. The use of non-medicinal parts will promote the comprehensive utilization of medicinal plants and reduce resource waste. However, there is limited research on the antibacterial active substances in the leaves of *F. suspensa*, and the antibacterial mechanism is not clearly defined, which limits their applications. Therefore, a stable and effective method for extracting antibacterial active ingredients from *F. suspensa* leaves using macroporous adsorption resin (MARs) was developed, and the antibacterial mechanism was discussed (Figure 1). The development and utilization of resources of non-medicinal parts of traditional Chinese medicine is a sustainable concept. The full use of *F. suspensa* leaves will improve production efficiency and avoid environmental pollution.

## Experimental Section

### Instruments and Materials

#### Instruments

The UPLC/Q-TOF MS analyses were performed on an AB SCIEX Triple-TOFTM 5600^+^ mass spectrometer and ExionLC AD (AB Sciex, MA, USA). The HPLC analyses were performed on Shimadzu 2010 CH HPLC system (Shimadzu, Japan). The vertical automatic high-pressure steam sterilization pot was used for the bacteria experiment (LDZX-75KB, Shanghai Shenan Medical Instrument Factory, China). Nitrogen blowing was carried out on a CM-12J type nitrogen blowing instrument (Beijing Chengmeng Weiye Technology Co., Ltd., China). A scanning electron microscope (SEM) Hitachi S-3400N equipped with an X-radiation detector EDS (Voyager of noran instruments). Gold thin films and nanoparticles were prepared by an ion coater (Eiko IB3, Tokyo, Japan). UV-visible spectrometry and multi-function microplate reader were used (TECAN Corporation, Austria). Reverse transcription polymerase chain reaction (RT-PCR) amplifications were performed on LineGene 9660 model FQD-96A (Hangzhou Bori Technology Co., Ltd., China).

#### Chemical Regents

AB-8 MARs were purchased from Suzhou Donghong Chemical Co., Ltd (Suzhou, China). HPLC-grade ethanol, methanol, and acetonitrile were purchased from Merck (Merck, Germany). Gentamicin sulfate injection was purchased from Henan Runhong Pharmaceutical Co., Ltd (Henan, China). 2.5% EM grade glutaraldehyde was purchased from AGAR Scientific (AGAR, United Kingdom). TRIzol™ reagent was purchased from Thermo Fisher Scientific (Thermo, United States). Water was purchased from Guangzhou Watsons Food & Beverage Co., Ltd (Guangzhou, China) and phosphate-buffered saline (PBS) was purchased from Invitrogen Co. Carlsbad (Invitrogen, United States). Dimethyl sulfoxide (DMSO) was purchased from Sigma-Aldrich Company Ltd. (St. Louis, United States). Forsythiaside A, forsythin, chlorogenic acid, and rutin standard (≥98% purity, HPLC) (National Institute for the Control of Pharmaceutical and Biological Products, China). Reverse transcription kit and Real-Time PCR kit were purchased from Takara Biotechnology Co., Ltd (Takara, China) and an alkaline phosphatase assay (AKP) Kit was purchased from MyBioSource, Inc (MyBioSource, United States). Mueller-Hinton (MH) agar medium, MH broth medium, and Lysogeny broth (LB) broth were prepared according to the manufacturer’s instructions and purchased from Qingdao Gaokeyuan Haibo Biotechnology Co., Ltd (Qingdao, China).

#### Bacterial Strains

The *Escherichia coli* (*E. coli*) O157:H7 (CICC 10899) and *Staphylococcus aureus* (*S. aureus*) (CICC 21601) strains were obtained from the China Center of Industrial Culture Collection (Beijing, China).

#### Plant Material

The *F. suspensa* leaves were collected in May 2019 from Tunliu County, Shanxi Province, and identified as *F. suspensa* leaves by Jinhai Huo (Heilongjiang Provincial Academy of Traditional Chinese Medicine, Harbin city, China). Finally, the leaves were picked and dried in a ventilated place.

### Adsorption of Phenethyl Alcohol Glycosides on the Resins

We modified a method of Sun partially and obtained a stable method for enriching phenethyl alcohol glycosides in leaves (Jian-rui et al., 2017). A total of 50.0 g leaves were accurately weighed and immersed in 600 ml water for 2 h, then reflux extraction was performed twice for 2 h each time. The extracts were combined in a rotary evaporator and evaporated to dryness, and then reconstituted with water to obtain 50 ml of sample solution. The MARs were pretreated by soaking in 95% ethanol overnight and then washed with water thoroughly to remove ethanol completely. A 4.2 cm × 100 cm glass column filled with 500 g AB-8 resin, which was used in the gradient elution tests with the flow rate is two bed volumes (BV)/h. Then the adsorbate-laden column was first washed with water and eluted with ethanol-water solutions of 10, 30, 50, and 70% in succession (represented by A, B, C, D, and E, respectively). The elution solution from each gradient elution solution was collected to the rotary evaporator and evaporated to dryness and dissolved in 100 ml water.

### HPLC, and UPLC/Q-TOF MS Analysis

**Sample preparation for HPLC:** The forsythiaside A standard was dissolved in methanol to prepare a sample with a concentration of 0.106 mg/ml. The A, B, C, D, and E elution were concentrated 5 times and filtered to obtain the test solution (0.45 μm water filter membrane (Tianjin Jinteng Experimental Equipment Co., Ltd.)). HPLC performance parameters were in the *Supplementary Material*.

**HPLC analysis:** The chromatographic system is equipped with a quaternary gradient pump, online degasser, auto-sampler, column thermostat, and Shimadzu liquid chromatography workstation. The chromatographic separation was performed on an Agilent ZORBAX C_18_ column (4.6 mm × 250 mm, 5 μm). Methanol-0.8% glacial acetic acid was used as the mobile phase, the flow rate was 0.8 ml/min. The detection wavelength was 280 nm, the injection volume was 10 μL, and the column temperature was 30 °C. The solvent gradient adopted was as follows: 0–6 min, 85%–74% B; 6–20 min, 74% B; 20–25 min, 74%–70% B; 25–30 min, 70%–63% B; 30–45 min, 63% B; 45–60 min, 63%–61% B.

**Sample preparation for UPLC/Q-TOF MS:** Standards of forsythin, chlorogenic acid, and rutin were dissolved in methanol solution to prepare a sample with a concentration of 95, 304, 138 μg/ml. The C elution solution (FC) was diluted 5 times and filtered (0.22 μm water filter membrane (Tianjin Jinteng Experimental Equipment Co., Ltd.)).

**UPLC/Q-TOF MS analysis:** The chromatographic separation was performed on a Waters ACQUITY UPLC BEH C_18_ column with 1.7 μm, 2.1 mm × 100 mm, and equipped a BEH C_18_ VanGuard Pre-Column with 1.7 μm, 5 mm × 2.1 mm, from the same supplier. The composition of the two mobile phases was 0.1% (v/v) formic acid in water (A) and acetonitrile (B): 0–5 min, 5%–25% B; 3–13 min, 25%–80% B; 13–23 min, 80%–100% B; 23–23.1 min, 100%–5% B; 23.1–25 min, 5% B. The separations were performed with a constant flow rate of 300 μL/min. The column oven and tray cooler temperatures were set to 30 and 4°C, respectively. 5 μL of samples were injected in a full loop injection mode. The electrospray MS detection was performed in positive and negative mode detection mode with the ion source voltage set to 5.5 kV/4.5 kV. Nitrogen was used as the sheath gas and helium as auxiliary gas with a flow rate of 10 and 0 arbitrary units, respectively. The mass spectrum scanning range is 80–1500 Da. The 80 V declustering potential, 35 eV/-35 eV collision energy, and 15 eV/-15 eV collision energy spread were used in mass detection. PeakView 2.0 was used to load published compounds into the MasterView function block for analysis.

### Antibacterial Potential

A standard agar disc diffusion method was used for the antibacterial assay. A standardized inoculum (100 µL) containing 10^7^ CFU/ml bacterial suspension was prepared by diluting an overnight culture in LB broth, and then spread evenly on the LB agar plate. Filter paper (6 mm diameter) was impregnated with 20 µL of FC and gentamicin sulfate injection (0.16 mg/ml) placed on inoculated agar, and incubated at 37 °C for 24 h. The antibacterial activity was evaluated by measuring the diameter of the inhibition zone against the tested bacteria.

The minimum inhibitory concentrations (MICs) and minimum bactericidal concentrations (MBCs) of FC were determined by the method of two-fold broth micro-dilution (Mellegård et al., 2011). The strains with a concentration of 10^7^ CFU/ml (100 uL) were added to each well of a 96-well plate containing 100 uL of serially diluted FC in MH broth. After incubating at 37 C with shaking for 24 h, the MICs were obtained by measuring the optical density (OD) at 600 nm. Furthermore, the aliquots from wells displaying no growth were spread on nutrient agar plates and incubated at 37°C for 24 h. Compared with the initial bacterial inoculum, the lowest concentration of the sample solution in the well that reduced the growth of bacteria on the agar plate by 99.9% was expressed as MBC.

The viable counts of tested bacteria were determined based on J Han’s detection method (Han et al., 2017). The FC at the MIC level was used to inoculate a bacterial suspension containing 10^7^ CFU/ml and incubate at 37°C with a 150 r/min shaking incubator for viable bacteria count. At 0, 20, 40, 60, 90, 120, 150, 210, and 300 min, 50 µL of bacterial suspension was applied to the surface of the MH nutrient agar which incubates in a 37°C incubator for 24 h and counts. 10^7^ CFU/ml text bacterial suspension was mixed with the FC to make the final concentration of MIC, and cultured at 37°C, 150 r/min shakers, with sterile water as a blank group. The OD value of the solution at 600 nm was measured by a spectrophotometer every 2 h (Filippini et al., 2011).

### Antibacterial Mechanisms

**Effect on morphological changes:** The state of the bacterial cell membrane treated with FC was observed by SEM. *S. aureus* and *E. coli* were respectively inoculated on MH and LB agar containing FC (at concentrations of both MIC and 2MIC), and cultured in a 37 °C incubator for 24 h. The specimens were collected and fixed in 2.5% (v/v) glutaraldehyde for 8 h, and then continuously exposed to ethanol in a concentration range of 30–100% to dehydrate the samples. The ethanol was replaced by 100% tertiary butyl alcohol. The specimens were dried with CO_2_ and the dried cells were coated with gold in a sputter coater after dehydration.

**Effect on cell wall permeability:** According to Juan Xie’s method, the AKP kit was used to detect cell wall permeability (Qiang et al., 2014). Each 10 ml centrifuge tube containing a bacterial suspension (approximately 10^5^ CFU/ml) in 2 ml of LB broth was inoculated with the MIC level of FC and incubated at 37°C and 150 r/min in a shaking incubator. In addition, the sample was replaced with sterile water as a blank group. After mixing and culturing, 1 ml of the bacterial suspension was centrifuged at 3,500 r/min for 10 min. The absorbance of the supernatant was measured every 2 hs at 680 nm by a microplate reader.

**Effect on membrane integrity:** The FC at the MIC level was added to the tube with the bacterial inoculum. The cells were centrifuged at 3,500 r/min after 0, 60, 120, 180, 240, 300, and 360 min of treatment, and the absorbance of the obtained supernatant was determined at 260 nm by a microplate reader (Xiao-fang et al., 2016).

**Effect on membrane efflux pump gene expression: Total RNA samples were isolated** from the bacterial cells using the TRIzol™ reagent (Monteiro et al., 2016). Reverse transcription of RNA was performed according to manufacturer instructions of the TaKaRa Reverse transcription Kit. The relative expression of bacterial cell membrane efflux pump genes was determined by the 2^−ΔΔCt^ method using quantitative RT-PCR (Livak and Schmittgen, 2001). Approximately 2 µg RNA (>200 nt) was reversely transcribed for cDNA synthesis by TaKaRa Reverse Kit. The primer sequences were as follows: 16sRNA (forward primer: 5′-CTC​CTA​CGG​GAG​GCA​GCA​G-3′, reverse primer: 5′-GWATTACCGCGGCKGCTG-3′), acrA (forward primer: 5′-ATC​GCA​GAA​GTT​CGT​CCT​CAA​GTT​AG-3′, reverse primer: 5′-ATC​GCA​GAA​GTT​CGT​CCT​CAA​GTT​AG-3′), and norA (forward primer: 5′-TCT​TGC​TAG​GTA​GTG​TAT​CGT​CTG​GAG-3′, reverse primer: 5′-CTT​GTA​TGG​AGG​CGG​CTT​GAC​C-3′). PCR reaction conditions are summarized in Table 1:

**TABLE 1:** RT-PCR reaction conditions.

Cycle	Step	Conditions	No. of cycles
1	Pre-denaturation	95 C for 30 s	1
2	Denature	95 C for 5 s	40
	Anneal/Collect Date	60 C for 30 s	
	Extend	72 C for 30 s	
3	Dissociation	72–95°C for 10 s	1

After the reaction, the RT-PCR amplification curves and splitting curves were observed and derived, and the Ct values of each group were output, and the reverse transcription polymerase chain reaction products were analyzed.

### Statistical Analysis

All the assays were carried out in triplicate and all the data were recorded as mean ± SD.

## Results and Discussion

### Analysis by HPLC and UPLC/Q-TOF MS

**FIGURE 2:**
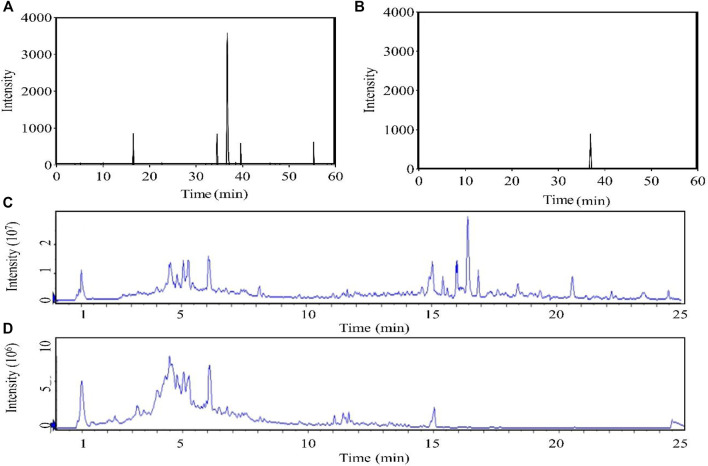
Chromatogram **(A)**: HPLC chromatogram. **(B)**: HPLC chromatogram of forsythiaside A standard. The total ion chromatogram obtained in the positive **(C)** and negative **(D)** modes.).

The content of forsythiaside A in *F. suspensa* leaves was determined by HPLC (Figures 2A,B). The content of B, C, D, and E elution solution was 73.93, 655.82, 18.34, and 12.37 mg/ml, respectively while there was almost no forsythiaside A in A elution solution. The results showed that the method could effectively enrich the active components of phenethyl alcohol glycosides in leaves.

The chromatographic conditions and mass spectrometry conditions were optimized for analysis and displayed the total ion chromatogram (Figures 2C,D). 31 compounds were identified including 11 glycosides, eight flavonoids, three organic acids, three phenolic, three lignans, one phenylpropanoid, one terpenoid, and one other substance (Table 2). According to the results of UPLC/Q-TOF MS and HPLC, the main components of FC were glycosides and flavonoids, while the component with antibacterial activity in glycosides was phenylethanoid glycosides. Forsythoside A was used as the component with the highest content. The MS^2^ chromatogram and product ion spectrum of forsythiaside A is shown in Supplementary Figure S1. ABDE fraction did not show antibacterial activity due to the low content of active ingredients of phenylethanoid glycosides.

**TABLE 2:** Components of FC of *F. suspensa* leaves.

No	RT (min)	Extracting ions	Measured mass (m/z)	Error (ppm)	Formula	Main secondary fragment ions (MS/MS) and sources	Identity	Category	Cas no
1	1.2	[M + H]^+^	123.0563	8.9	C_14_H_20_O_8_	317, 299, 281, 263, 227, 217, 203, 161	4-hydroxy-4-[2-[(2S,3S,4R,5R,6S)-3,4,5-trihydroxy-6-(hydroxymethyl) oxan-2-yl] oxyethyl] cyclohexa-2,5-dien-1-one	Glycosides	40661–45–8
2	3.4	[M + H]^+^	155.0697	3.7	C_8_H_10_O_3_	155, 109, 96, 95, 91	3a-hydroxy-2,3,7,7a-tetrahydro-1-benzofuran-6-one	Other	94535–01–0
3	4.1	[M + H]^+^	361.1663	4.8	C_20_H_24_O_6_	361, 343, 315, 152, 134	Lariciresinol	Glycosides	27003–73–2
4	4.4	[M + H]^+^	127.0387	2.1	C_6_H_6_O_3_	127, 109, 81	Benzene-1,2,4-triol	Phenolic	533–73–3
5	5.1	[M + H]^+^	163.0750	2.2	C_10_H_10_O_2_	163, 131, 103	Safrole	Phenylpropanoid	94–59–7
6	5.3	[M + H]^+^	463.1232	0.6	C_22_H_22_O_11_	463, 355, 337, 309, 295, 205, 187, 169, 151, 133, 127	Tectoridin	Flavonoids	611–40–5
7	5.5	[M + H]^+^	355.1024	0.1	C_16_H_18_O_9_	355, 163, 145, 117	Chlorogenic acid	Organic acids	327–97–9
8	6.5	[M + H]^+^	521.2014	0.7	C_26_H_32_O_11_	521, 163,161, 137, 103	(3R,4R)-4-[(4-hydroxy-3-methoxyphenyl) methyl]-3-[[3-methoxy-4-[(2S,3R,4S,5S,6R)-3,4,5-trihydroxy-6-(hydroxymethyl) oxan-2-yl] oxyphenyl] methyl] oxolan-2-one	Glycosides	23202–85–9
9	7.9	[M + H]^+^	303.0500	0.2	C_15_H_10_O_7_	303, 285, 257, 229, 201, 165, 153, 137	Quercetin	Flavonoids	117–39–5
10	8.0	[M + H]^+^	465.1028	0.1	C_21_H_20_O_12_	465, 303, 285, 257, 229	Quercetin 3-β-D-glucoside	Flavonoids	482–35–9
11	8.6	[M + H]^+^	449.1082	0.8	C_21_H_20_O_11_	449, 287, 165	Kaempferol 3-O-glucoside	Flavonoids	480–10–4
12	8.6	[M + H]^+^	287.0552	0.7	C_15_H_10_O_6_	287, 241, 213, 185, 171, 165, 157, 121, 107	Kaempferol	Flavonoids	520–18–3
13	8.6	[M + H]^+^	479.1549	0.2	C_23_H_26_O_11_	479, 325, 263, 245, 163, 145, 117	Calceolarioside B	Glycosides	105,471–98–5
14	8.7	[M + H]^+^	181.0487	4.6	C_9_H_8_O_4_	181, 163, 145, 135	*Cis*-caffeic acid	Organic acids	331–39–5
15	11.5	[M + H]^+^	321.0606	0.3	C_15_H_12_O_8_	321, 303, 257, 229, 213, 169, 141, 123	Dihydromyricetin	Flavonoids	27200–12–0
16	11.6	[M + H]^+^	359.1486	0.9	C_20_H_22_O_6_	359, 341, 311, 205, 151, 137, 122	Terpineol	Lignans	8,006–39–1
17	14.4	[M + H]^+^	447.0925	0.1	C_21_H_18_O_11_	447, 271	6-(5,6-dihydroxy-4-oxo-2-phenylchromen-7-yl)oxy-3,4,5-trihydroxyoxane-2-carboxylic acid	Glycosides	100,647–26–5
18	5.1	[M-H]^-^	299.1130	2.1	C_14_H_20_O_7_	299, 165, 151, 137, 119	Salidroside	Glycosides	10338–51–9
19	5.1	[M-H]^-^	137.0246	1.3	C_7_H_6_O_3_	137, 119, 109	3,4-dihydroxybenzaldehyde	Phenolic	139–85–5
20	5.5	[M-H]^-^	389.1442	2.6	C_17_H_26_O_10_	389, 223, 181, 150	Loganin	Glycosides	18524–94–2
21	5.7	[M-H]^-^	537.1966	2.1	C_26_H_34_O_12_	537, 375, 345, 327, 297, 282, 279	(+)-8′-hydroxyariciresinol 4′-O-β-d-glucopyranoside	Glycosides	76880–93–8
22	6.5	[M-H]^-^	191.0561	0.1	C_7_H_12_O_6_	191, 127, 109	Quinic acid	Organic acids	36413–60–2
23	7.6	[M-H]^-^	609.1817	1.3	C_28_H_34_O_15_	609, 447, 419, 301, 271, 179, 161	Hesperidin	Flavonoids	520–26–3
24	7.8	[M-H]^-^	609.1467	1.0	C_27_H_30_O_16_	609, 301, 271, 255, 243, 178, 151	Rutin	Flavonoids	168,111–03–3
25	8.2	[M-H]^-^	755.2409	0.7	C_34_H_44_O_19_	755, 623, 593, 161	2-(((3,4-dihydroxy-4-(hydroxymethyl) tetrahydrofuran-2-yl) oxy) methyl)-6-(3,4-dihydroxyphenethoxy)-5-hydroxy-4-((3,4,5-trihydroxy-6-methyltetrahydro-2H-pyran-2-yl) oxy) tetrahydro-2H-pyran-3-yl (E)-3-(3,4-dihydroxyphenyl) acrylate	Glycosides	81525–13–5
26	8.6	[M-H]^-^	623.1978	0.5	C_29_H_36_O_15_	624, 461, 443, 179, 161	Forsythoside A	Glycosides	79916–77–1
27	16.1	[M-H]^-^	371.1499	0.3	C_21_H_24_O_6_	371, 356, 151, 136, 121	Arctigenin	Lignans	26687–82–1
28	18.4	[M-H]^-^	163.0763	1	C_10_H_12_O_2_	163, 148, 119	Engenol	Phenolic	97–53–0
29	23.1	[M-H]^-^	471.3475	1	C_30_H_48_O_4_	471, 407	Corosolic acid	Terpenoid	4,547–24–4
30	5.1	[M + Na]^+^	485.1625	0.9	C_20_H_30_O_12_	485, 339	Forsythoside E	Glycosides	93675–88–8
31	7.8	[M + Na]^+^	557.1945	8.6	C_27_H_34_O_11_	557, 395, 379, 309, 201, 185	Forsythin	Lignans	96420–61–0

### Assay of Antibacterial Potential

The *in vitro* antibacterial activity of FC against two bacteria was assessed based on the presence of inhibition zones (Table 3 and Supplementary Figure S2). The diameter of inhibition zone >15 mm was indicated high sensitivity, 10–14 mm was moderate sensitivity, 6–9 mm was low sensitivity, and 5.0 mm has no antibacterial effect.

**TABLE 3:** Inhibition zone diameter (n = 3).

Bacterial strains	Inhibition zone diameter (mm)				
	Concentration of FC (g/ml)			Forsythiaside a (5 mg/ml)	Gentamicin (0.16 mg/ml)
	0.05	0.10	0.50		
*S. aureus*	9.36 ± 0.10	12.22 ± 0.32	16.77 ± 0.26	17.88 ± 0.01	18.47 ± 0.13
*E. coli*	10.23 ± 0.15	14.41 ± 0.13	17.56 ± 0.12	18.14 ± 0.17	20.28 ± 0.13

The FC showed antibacterial activity against *S. aureus* and *E. coli*. The MIC of *S. aureus* and *E. coli* was 7.81 and 3.91 mg/ml, and the MBC was 7.81 and 15.63 mg/ml.

**FIGURE 3:**
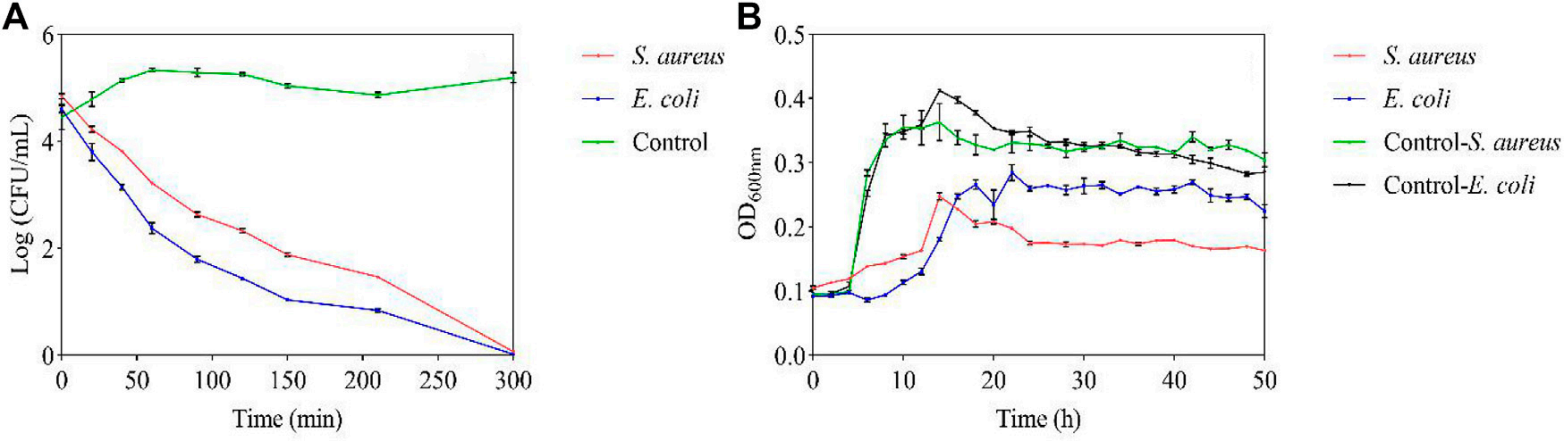
Antibacterial potential of FC at MIC **(A)**: Viable counts. **(B)**: Growth curve).

By measuring the viability of the tested bacteria, their survival during the 300 min with FC treatment was inhibited. The growth of the two tested bacteria was inhibited at the MIC, and 50% inhibition of cell viability was detected within 20 min. After 210 min, 80% bacterial inhibition was observed, and all tested bacteria were completely inhibited by FC after 300 min (Figure 3A). According to the experimental results, FC has an inhibitory effect on the growth of the tested bacteria (Figure 3B). The control group has typical growth curve characteristics. The tested bacteria were always lower than the control group during the whole growth period, indicating that the FC was against *S. aureus* and *E. coli*.

### Assay of Antibacterial Potential

**FIGURE 4:**
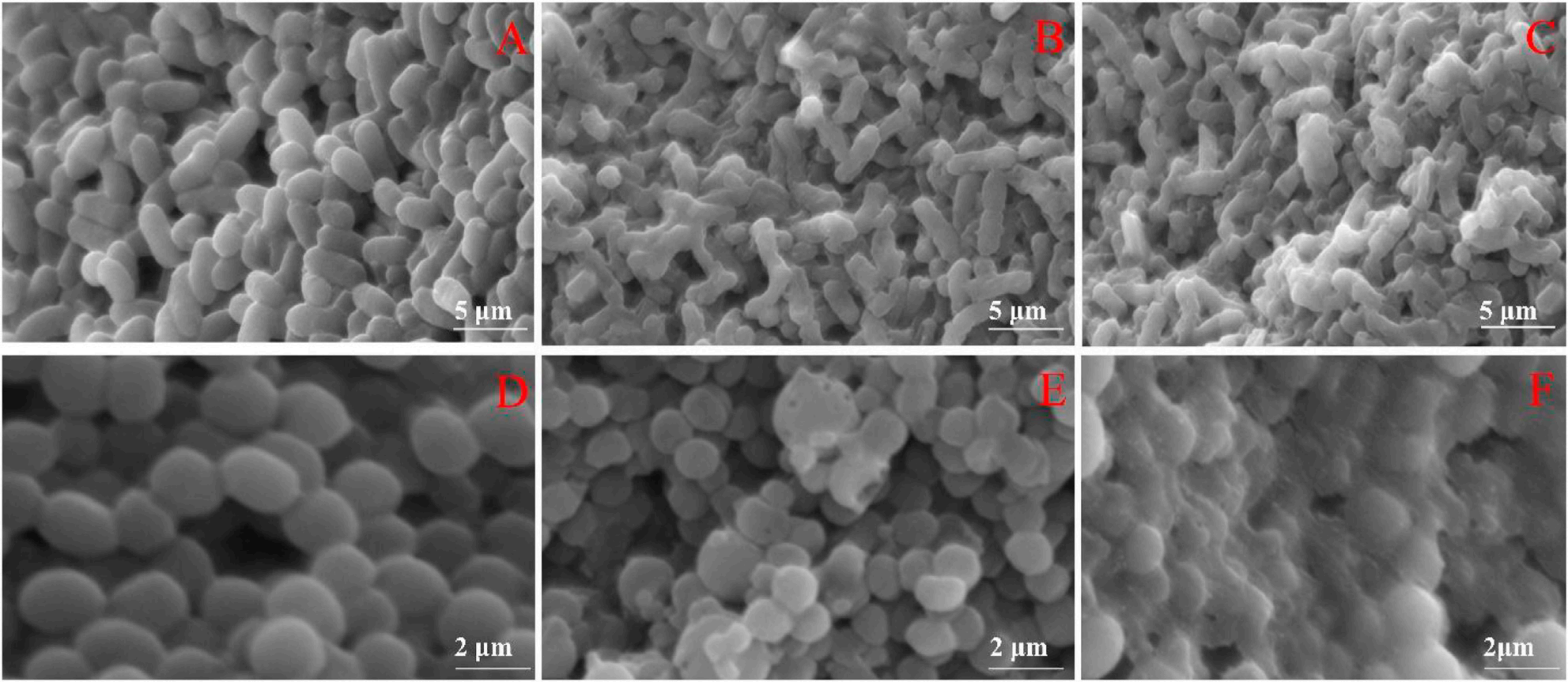
Effects of FC on the morphology of tested bacteria **(A)**: Untreated *E. coli*. **(B)**: *E. coli* treated with MIC. **(C)**: *E. coli* treated with 2MIC. **(D)**: Untreated *S. aureus*. **(E)**: *S. aureus* treated with MIC. **(F)**: *S. aureus* treated with 2MIC).

Through SEM, it was observed that *E. coli* in the blank group had a regular rod shape, intact surface. The shape of most *E. coli* treated with FC became irregular, and shrinked to varying degrees. The shape of *S. aureus* in the blank group was a regular spherical shape, with complete cells and a smooth surface. After *S. aureus* was exposed to FC solution, its cell membrane appears pitted, shrunk, holes appear on the surface, which may even cause the cell to rupture and the contents to flow out. The changes of test bacteria may be caused by the destruction of the membrane and the loss of intracellular substances in *E. coli* and *S. aureus* caused by FC. (Figure 4).

**FIGURE 5:**
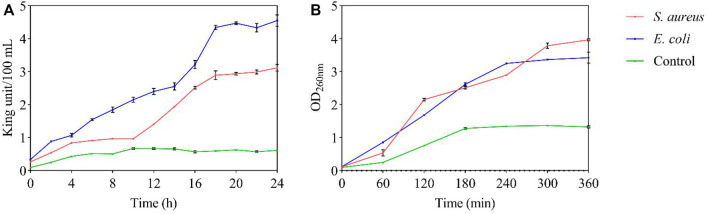
Antibacterial mechanism of FC at MIC on test bacteria **(A)**: Cell wall permeability; **(B)**: Membrane integrity).

AKP is an intracellular enzyme. Normally, its activity can not be detected outside the cell. However, when the cell wall or membrane is destroyed, the permeability is increased and then AKP leaks out of the cell (Bucekova et al., 2019). Such permeability change occurred in the presence of FC **(** Figure 5A **)**, therefore, we conclude that FC can destroy the cell walls of *S. aureus* and *E. coli*. Proteins and nucleic acids are extremely important macromolecules for cells, providing cellular structure, function, and genetic information. According to the method of Paul, the absorbance of nucleic acids and proteins at 260 nm could be an indicator of membrane integrity (Bajpai et al., 2013; Paul et al., 2011; Hao et al., 2020). With time, the release of cellular components increased significantly at MIC (Figure 5B). This result indicated that FC affected the integrity of the membrane, causing nucleic acids and proteins to leak through the membrane, leading to cell death. Another possibility is that FC may have penetrated the cell membrane and free radicals were produced, which oxidize and damage lipids, proteins, and DNA (Bakkali et al., 2008).

**FIGURE 6:**
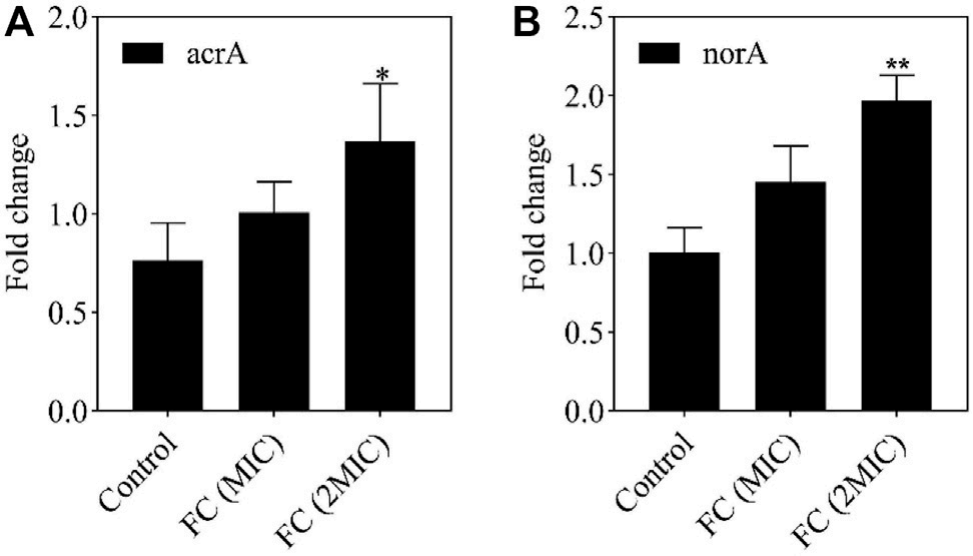
The effect of MIC and 2MIC concentration FC on gene expression levels. The fold change in the expression level of acrA **(A)**, norA **(B)** in the *E. coli*, *S. aureus* of different concentration (MIC, 2MIC) of FC group were compared to the control without FC treatment by using RT-PCR. Data from the RT-PCR analysis is represented as mean ± SEM. By *t*-test analysis, *p < 0.05, **p < 0.01 comparing to the control group.

An in-depth study of specific drug resistance mechanisms was outside the scope of this study. However, a targeted study of some drug delivery pumps with clear pump functions can be used to screen new antibacterial drugs and resistance inhibitors (Kaatz and Seo, 2004). The expression of the acrA gene can cause *E. coli* to be resistant to many drugs. The expression of the norA gene can cause *S. aureus* to be resistant to many drugs. Therefore, the expression of the active efflux gene acrA/norA plays a crucial role in the drug resistance of *E. coli*/*S. aureus*. The dissociation curve of RT-PCR indicated that the melting temperature of the amplification product is stable, the amplification product is single, the primer design is correct, there are no unspecific products, and the test results are accurate. As shown in Figure 6, the acrA and norA expression levels in the control group were lower than those in the FC treatment group. After different concentrations of FC treatment (MIC, 2MIC), the expression levels of acrA and norA proteins were up-regulated. We infer that the cellular damage caused by FC components can interfere with the development of drug resistance in these and possibly other bacteria. However, due to the complexity of the drug resistance mechanism, our team will conduct in-depth research on the antibacterial mechanism of *F. suspensa* leaves in the next step.

## Conclusion

In this paper, MARs were used to enrich the active components of *F. suspensa* leaves, which had the advantages of being fast, efficient, economical, and recyclable. Compared with the traditional HPLC analysis, this research used UPLC/Q-TOF MS for the first time to identify 31 compounds in the extract of *F. suspensa* leaves, which deepened the understanding of their chemical constituents. At present, the research on *F. suspensa* leaves mainly focuses on the extraction, isolation, and structural identification of individual components, without the overall analysis and in-depth study of its pharmacological effects and the antibacterial mechanism has not been clearly explained. In this article, the antibacterial effect of leaves and its antibacterial mechanism have entered in-depth research, including the determination of the inhibition zone diameter, MIC and MBC, the growth curve, viable counts, external morphology changes, permeability/integrity of the cell wall/membrane, and changes in the expression of a cell membrane efflux pump gene. Taken together, our findings provide evidence that the extract of F. suspensa leaves exhibited an antibacterial effect that involves damaging the bacterial cell membrane and raising bacterial cell wall permeability abnormally. This is conducive to more comprehensive utilization of medicinal plant resources.

## Data Availability

The raw data supporting the conclusions of this article will be made available by the authors, without undue reservation.
