# Supplementary material for: UPLC/Q-TOF MS Screening and Identification of Antibacterial Compounds in Forsythia suspensa (Thunb.) Vahl Leaves
Source: Front Pharmacol. 2022 Jan 28;12:704260. doi: 10.3389/fphar.2021.704260 (PMC8831367; doi:10.3389/fphar.2021.704260)
Supplement: Supplementary file 1 [file DataSheet1.docx]

Supplementary Material

# Supplementary Data

1.1 HPLC performance parameters

1.1.1 Standard linearity

A series of forsythiaside A standards will be prepared in triplicate at six different concentration levels, i.e. 0, 10%, 20%, 40%, 60% and 80% of the nominal assay concentration, corresponding to 0, 0.0106, 0.0212, 0.0424, 0.0636, 0.0848 mg/mL. The standard will be analyzed by the method and the linearity of the data will be determined by evaluating the slope, intercept, and correlation coefficient (r) of the linear regression line for the response versus the actual concentration plot.

1.1.2 Spiked forsythiaside A standard recovery

Triplicate FC solution will be prepared and spiked with forsythiaside A standard at the above-stated range of concentrations. These FC solutions will be evaluated for accuracy by comparison of the detected concentration versus the actual concentration of forsythiaside A standard for each sample.

1.1.3 Precision

Replicate injections (n=6) of a solution with a forsythiaside A standard will be performed and the % RSD for peak area response will be evaluated.

1.1.4 Stability

The forsythiaside A standard was injected at 0, 2, 6, 8, 12, 24 h, and the % RSD for peak area response will be evaluated.

1.1.5 Repeatability

Replicate injections (n=6) of a solution with FC solution will be performed and the % RSD for peak area response will be evaluated.

1.1.6 Specificity

Specificity refers to the ability to clearly evaluate the analyte that may be present in the component. The measured sample and methanol as a blank sample were injected and analyzed.

1.1.7 Determination of limit of detection/quantification

The measured sample is injected and analyzed. When the signal-to-noise ratio is 10, this concentration is the limit of quantification. When the signal-to-noise ratio is 3, this concentration is the detection limit. The signal-to-noise ratio means signal/noise.

1.2 Result

1.2.1 Standard linearity result

The y-intercept of the plot of peak area response versus concentration is not significantly different from 0 at the 95% confidence level (t(intercept) ≤ t(tabular)). The correlation coefficient (r) is 0.99. The final linear equation of forsythiaside A is Y=1380512534x+13486 (R^2^=0.999), and the linear range is 0.02～0.16 mg/mL.

1.2.2 Spiked forsythiaside A standard recovery result

The average percent recovery is 95 to 105 ％ of the theoretical value (% RSD=2.17).

1.2.3 Precision result

% RSD (n=6) =0.41(≤2) at 100% of the nominal assay concentration.

1.2.4 Stability result

% RSD (n=6) =0.026 (≤2) at 100% of the nominal assay concentration.

1.2.5 Average content repeatability result

% RSD (n=6) =0.12 (≤2) at 100% of the nominal assay concentration.

1.2.6 Specificity

According to the HPLC results, the existence of forsythiaside A can be clearly distinguished in Figure 2A-B.

1.2.7 Limit of Detection/Quantification

The limit of quantification is 2.35 mg/mL and the limit of detection is 0.65 mg/mL.

# Supplementary Figures and Tables


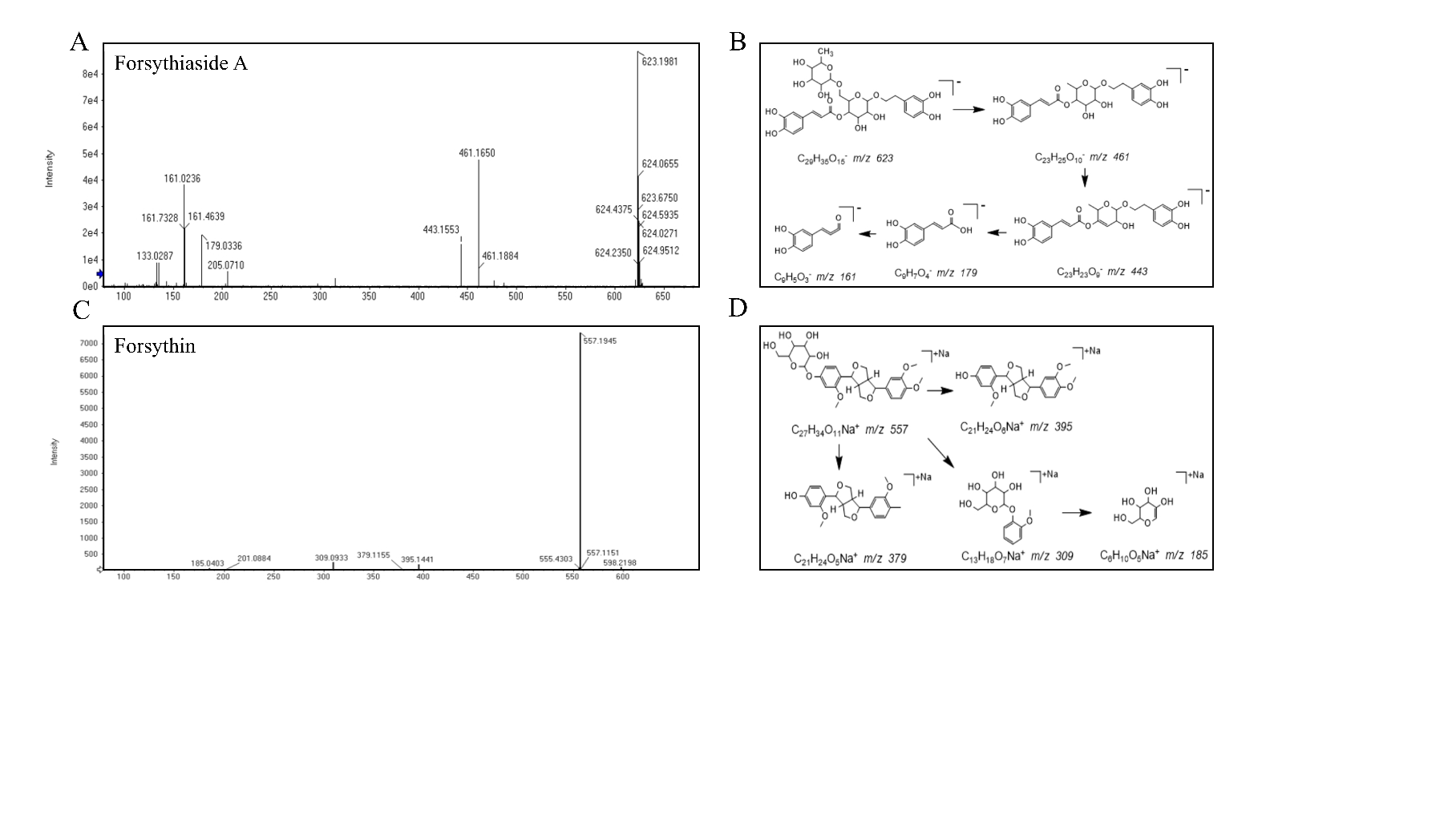


**Figure S1** MS^2^ chromatogram of forsythiaside A (A). The product ion spectra of forsythiaside A (B).


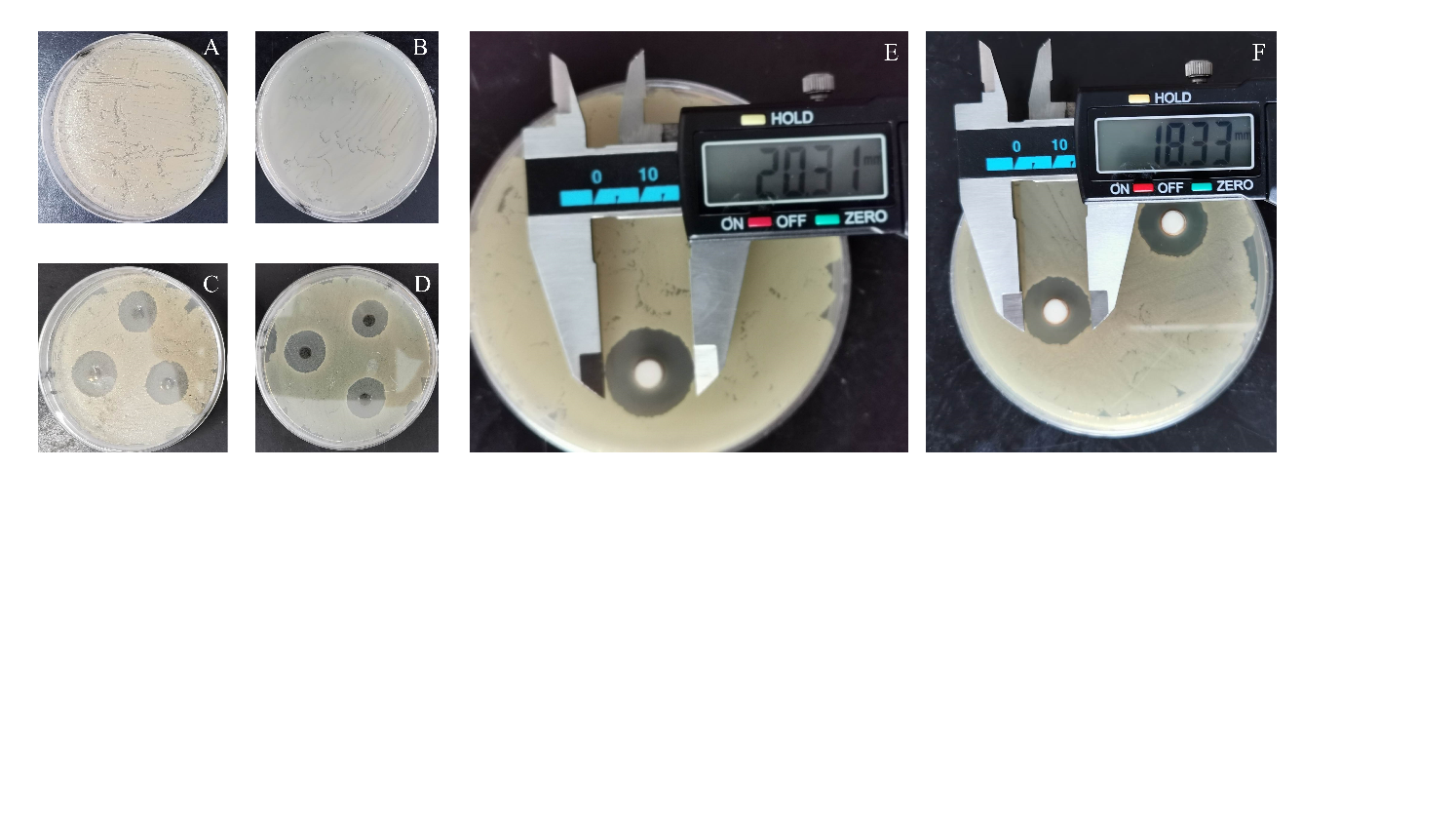


**Supplementary Figure S2.** The Inhibition zone diameter (A: *S. aureus*. B: *E. coli*. C: *S. aureus* treated with different concentrations of FC. D: *E. coli* treated with different concentrations of FC. E: *E. coli* treated with Gentamicin (0.16 mg/mL). F: *S. aureus* treated with Gentamicin (0.16 mg/mL))
